# Mechano-Chemical Aspects of Organ Formation in *Arabidopsis thaliana*: The Relationship between Auxin and Pectin

**DOI:** 10.1371/journal.pone.0057813

**Published:** 2013-03-12

**Authors:** Siobhan A. Braybrook, Alexis Peaucelle

**Affiliations:** 1 Institute of Plant Sciences, University of Bern, Bern, Switzerland; 2 Laboratoire Matière et Systèmes Complexes, UFR de Physique, Université Paris Diderot, Paris, France; 3 Institut Jean-Pierre Bourgin, UMR1318 INRA-AgroParisTech, INRA Centre de Versailles-Grignon, Versailles, France; Umeå Plant Science Centre, Sweden

## Abstract

How instructive signals are translated into robust and predictable changes in growth is a central question in developmental biology. Recently, much interest has centered on the feedback between chemical instructions and mechanical changes for pattern formation in development. In plants, the patterned arrangement of aerial organs, or phyllotaxis, is instructed by the phytohormone auxin; however, it still remains to be seen how auxin is linked, at the apex, to the biochemical and mechanical changes of the cell wall required for organ outgrowth. Here, using Atomic Force Microscopy, we demonstrate that auxin reduces tissue rigidity prior to organ outgrowth in the shoot apex of *Arabidopsis thaliana*, and that the de-methyl-esterification of pectin is necessary for this reduction. We further show that development of functional organs produced by pectin-mediated ectopic wall softening requires auxin signaling. Lastly, we demonstrate that coordinated localization of the auxin transport protein, PIN1, is disrupted in a naked-apex produced by increasing cell wall rigidity. Our data indicates that a feedback loop between the instructive chemical auxin and cell wall mechanics may play a crucial role in phyllotactic patterning.

## Introduction

Patterns in nature have always fascinated humans, from children to scientists. As exemplified by the seminal work of Alan Turing [1], scientists of diverse disciplines have all attempted to explain biological patterns within their own frameworks [2]. Within the field of developmental biology, these disciplines have been interacting more and more to provide richer details for patterning mechanisms, a trend which will surely continue [3], [4], [5]. One of the most riveting proposals of Turing is that models explaining morphogenesis should consist of 'two parts, the mechanical and the chemical' [1], [4]; using this simple statement as a starting point, we have undertaken to examine how a chemical signal, it's chemical responses, and it's mechanical outputs combine in plant patterning to provide a mechano-chemical regulatory loop.

The pattern of aerial organs in plants, or phyllotaxis, is highly regulated. Within the past ∼10 years a picture has emerged of the instructive mechanism for phyllotaxis: regulated distribution and accumulation of the phytohormone auxin [6], [7]. Through a series of biological and computational approaches it has been demonstrated that the correct distribution of auxin by its efflux transport proteins, the PIN family, is necessary and sufficient (*in silico*) for the establishment of phyllotactic patterns [8], [9], [10], [11], [12], [13]. The emergence of new organs, once positioned by auxin, requires precisely regulated cell expansion. Since cell expansion is mechanically limited by the cell wall, organ emergence ultimately requires changes in the cell wall chemistry or structure that then affect its mechanical properties. A large and historically rich body of evidence indicates that auxin can induce changes in the cell wall mechanical properties, largely through supposed acidification of the cell wall compartment [14], [15]. The acidification of the cell wall is thought to trigger enhanced activity of several wall modifying agents leading to enhanced elastic and viscoelastic behaviors (for review see [16]). In shoot apices, the wall modifying agent expansin has been demonstrated to trigger organ formation [17], [18], [19], and the alteration of pectin de-methyl-esterification in cell walls is necessary and sufficient for organ formation in wild-type apices [20]; however, observations of auxin induced changes in cell wall mechanics in the shoot apex have remained elusive. Recent work also suggests that regulated auxin transport may be effected either by tissue mechanics 21,22, by the mechanical integrity of the cell wall itself [23], or by mechanical strain in the cell wall and membrane ultimately affecting auxin transporter delivery [24] - implying the existence of a mechano-chemical regulatory loop in plant organ development.

Within this work we will focus on a particular mechanical property of the cell wall, elasticity (hereafter referred to as its converse, rigidity), its regulation by auxin, and how it relates to organ growth. The relationship between cell wall rigidity and cell growth is correlative at best. There is a body of work indicating that auxin affects rigidity of plant tissues [25], [26], [27], and there are numerous examples of correlations between tissue rigidity and growth [25], [28], [29], [30], [31], [32], [33], [34], [35]. The closest we have come recently to direct evidence of elasticity effecting growth lies in manipulating the chemistry of the pectin matrix, effecting rigidity, and seeing changes in organ emergence in the apex [34]. Indeed, even here there is debate: how could the cell wall matrix control growth when we know that cellulose fibers are the load bearing component of the wall? Interestingly, there is a wealth of evidence pointing to a role for pectins in plant growth although the idea has been limited to the field of algal growth [33] or lost in history 26,36,37,38. We have recently discussed several possible ways that changes in the mechanical properties of the pectin matrix could alter higher plant growth [32]. In the following work, we focus on further exploring the idea that changes in the pectin matrix, and cell wall elasticity, are essential for growth in plants.

Within this work, we use Atomic Force Microscopy on living *Arabidopsis* meristems to study the relationship between auxin signaling, pectin de-methyl-esterification, and cell wall rigidity. We demonstrate that auxin induces a reduction in cell wall rigidity at the shoot apex. We show that this process strictly requires de-methyl-esterification of the pectin homogalacturonan (HG), and that inhibition of HG de-methyl-esterification disrupts organized polarity of the auxin transporter PIN-FORMED1 (PIN1). We also demonstrate that while de-methyl-esterification of pectin alone is sufficient to induce local tissue growth in the meristem, auxin signaling is required for the formation of a fully structured organ- supporting the presence of a mechano-chemical regulatory loop between auxin and organ outgrowth.

## Results

### Auxin induces a decrease in cell wall rigidity prior to organ emergence

**Figure 1:**
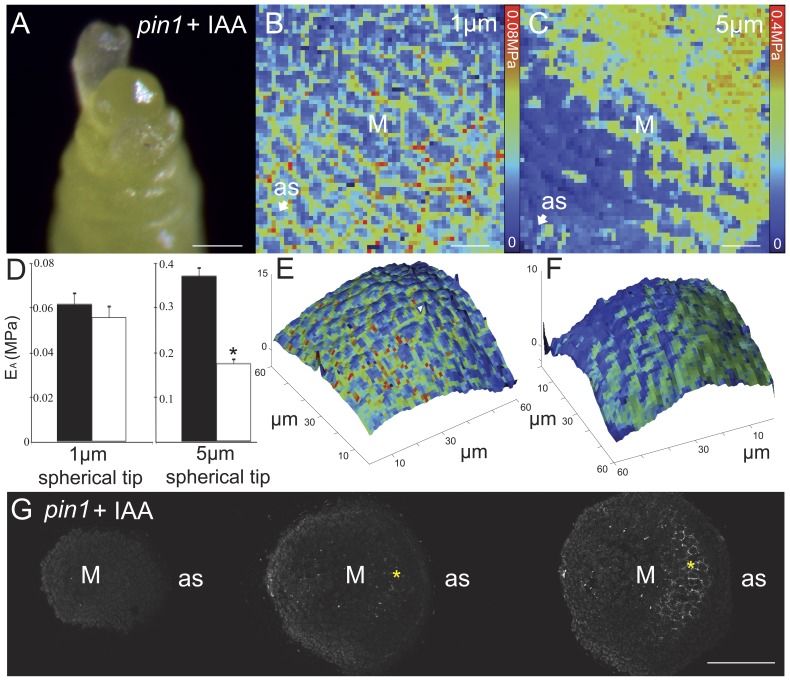
IAA application on *pin1* meristem leads to local tissue softening and pectin de-methyl-esterification in sub-epidermal tissues. (A) IAA induced organ formation in a *pin1* mutant inflorescence apex (t = 72 h post application). Apparent Young's modulus (E_A_, or 'rigidity') map of a representative *pin1* meristem ∼18 hours post IAA application as determined with a 1 µm (B) or 5 µm (C) spherical tip. Total number of meristems analyzed + IAA, n = 13. Each pixel in a rigidity map corresponds to the E_A_ value obtained from one indentation point. (D) Graphical display of averaged E_A_ data from all meristems with values for meristem (black bars) and just above application site (white bars). Significant difference indicated by asterisk at p-value<0.01 (T-test on averages from n meristems: ‘*pin1*–IAA’ 5 µm n = 6 and 1 µm n = 7 (p-values 0.71 and 0.57 respectively), ‘*pin1*+IAA’ n = 13 (p-values: 1 µm p = 0.02, 5 µm p = 2.2E-5). Error bars are propagated standard deviations). Non-averaged results for all meristems can be found in Figure S1 (displaying reduced rigidity: 1 µm, +Inactive IAA n = 0/7, +IAA n = 1/13. 5 µm, +Inactive IAA n = 3/7, +IAA n = 13/13). (E,F) Topographical reconstruction of measured surfaces, as estimated by AFM point-of-contact, with the rigidity maps of (B,C) respectively used to color the surface. Note that meristem curvature does not correlate with areas of decreased E_A_, and that there is no bulging of the meristem accompanying decreased rigidity. (G) Serial transverse sections showing 2F4 labeling of HG de-methyl-esterification in a representative *pin1* meristem ∼18 hours after IAA application (n = 9). M: meristem, as: application site, Scale bars  = 100 µm (A,G) or 10 µm (B,C). Asterisk in (G) indicates 2F4 labeling in sub-epidermal tissues. Statistics in Figure S2, control data in Figure S3.

Previous observations have demonstrated that cell walls in the Arabidopsis inflorescence shoot apex displayed a reduced rigidity at emerged and incipient organ sites [34]. Based on the wealth of knowledge surrounding the role of auxin in organ positioning and emergence, we investigated whether auxin was sufficient to trigger a reduction in cell wall rigidity (measured as a reduction in cell wall apparent Young's modulus (E_A_) [34]). We used an auxin efflux carrier mutant, *pin1*, which displays an organ free apex [39], as a template to examine auxin induced changes in wall mechanics. As shown previously, local application of the natural auxin Indole Acetic Acid (IAA) rescued organ formation, with bulges becoming visible after ∼24–30 h and full organs after 72 h (Fig. 1A, [8]); bulging was defined as an AFM-detectable change in surface topology. Interestingly, a decrease in cell wall rigidity was observed surrounding the position of auxin application as early as 18 h post application, before any detected bulging (Fig. 1C, 1D, 1F, Fig. S1, Fig. S2, Fig. S3). Note that Figure S4 diagrams the application site relative to the area analyzed by AFM. In order to assess the bias introduced by sample curvature, fake silicon *pin1* meristems were produced which would mimic sample geometry, but have uniform rigidity (for further details see Technical Discussion). The analysis and comparison of representative samples may be found in Figure S5; in summary, geometrical bias was minimal compared to biological changes induced by IAA.

As previously shown in wild type incipient organ sites [34], rigidity changes were only observed with AFM tips loaded with 5 µm spherical tips (n = 13/13) and not with 1 µm spherical tips (Fig. 1B, 1D, 1E vs. 1C, 1D, 1F, Fig. S2, Fig. S3); there is a strong possibility that this discrepancy reflects changes occurring first in subepidermal tissues or non-surface walls, a hypothesis also supported by chemical and genetic data [34]. Neither mechanical changes nor organ formation occurred when inactivated IAA was applied to *pin1* apices (Fig. S3). This data provides direct evidence for measurable changes in cell wall rigidity, after auxin application in shoot apices, which presage organ outgrowth.

### Auxin induces local de-methyl-esterification of HG in subepidermal tissues

Since organ formation was previously shown to be dependent on the de-methyl-esterification of HG [20], [34], we next analyzed the HG methyl-esterification status in *pin1* apices following IAA application using immunolocalization of de-methyl-esterification by the anti-body 2F4. We observed 2F4 labeling locally below the auxin application site (Fig. 1G), whereas no such changes were observed for the mock-treated samples (Fig. S3). 2F4 labeling was only detected in subepidermal tissues, consistent with a proposed decreased rigidity in deeper tissue layers. These observations were also consistent with observed 2F4 labeling in wild-type shoot apices [34]. These data indicate that auxin acts, in part, by triggering the de-methyl-esterification of HG, causing a decrease in wall rigidity.

### Inhibition of HG de-methyl-esterification blocks auxin induced organ formation

**Figure 2:**
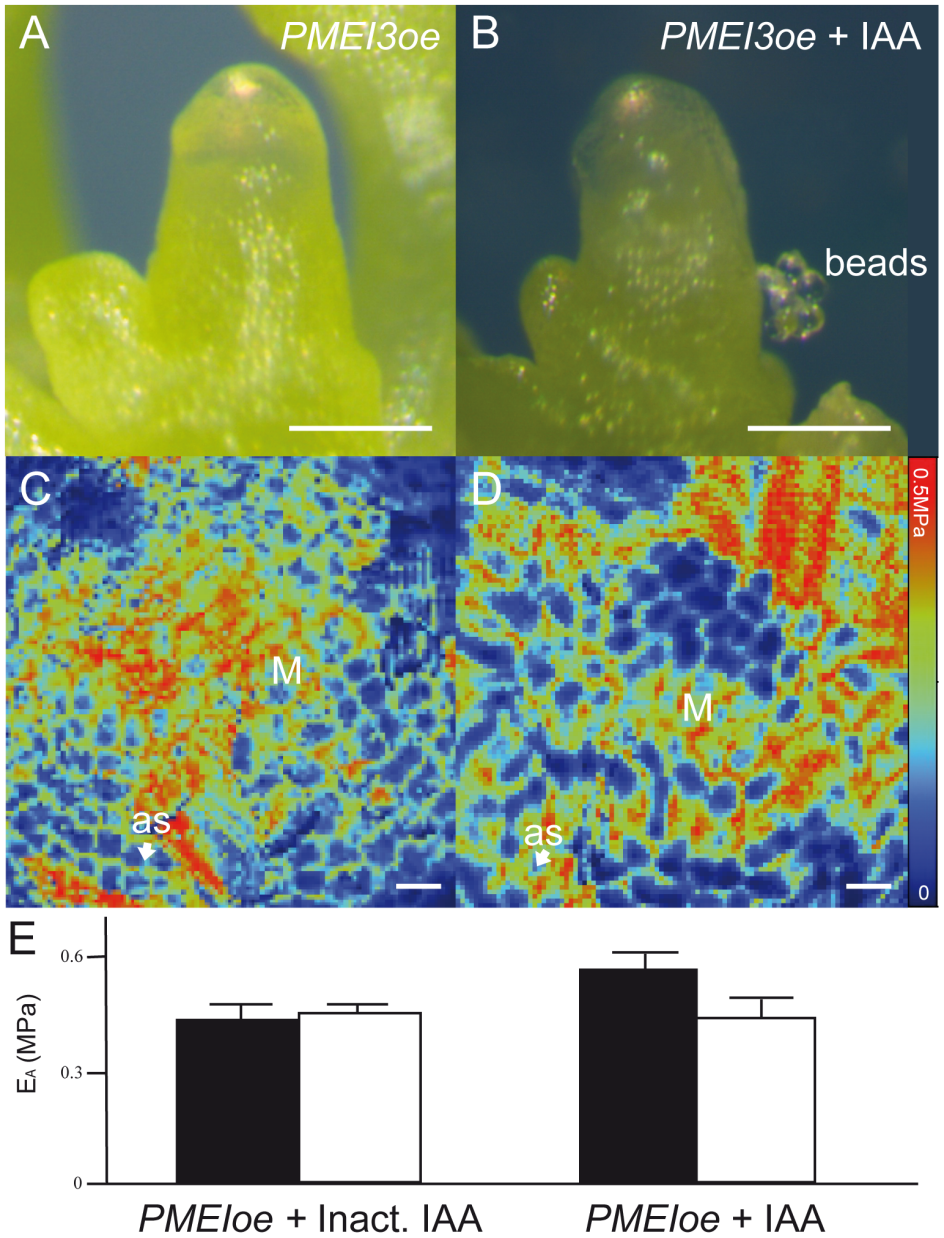
Blocking pectin de-methyl-esterification inhibits IAA-induced organ formation and tissue softening. (A)Representative *PMEI3oe* meristem ∼24 hours after PMEI induction. (B) Representative induced *PMEI3oe* meristem ∼72 hours post IAA application. (C,D) Apparent Young's modulus (E_A_, or 'rigidity') map of a representative control (C) or IAA applied (D) *PMEI3oe* meristem ∼18 hours after treatment as visualized with a 5 µm spherical tip. Analyzed meristems: control (n = 11), +IAA (n = 9). (E) Graphical display of averaged E_A_ data from all meristems with values for meristem (black bars) and just above application site (white bars). No significant difference was found in either treatment or control (T-test on averages from n meristems: *PMEI3oe* -IAA n = 9, meristem vs. periphery p-value  = 0.037; *PMEI3oe* +IAA n = 11, meristem vs. periphery p-value = 0.098. Error bars are propagated standard deviations. Statistics in Fig. S2); both showed higher variability than non-transgenic meristems. Non-averaged results for all meristems can be found in Figure S6 (displaying reduced rigidity: +Inactive IAA n = 1/11, +IAA n = 2/9). M: meristem, as: application site, Scale bars  = 100 µm (A,B) or 10 µm (C,D).

To confirm that HG de-methyl-esterification takes place downstream of auxin accumulation during organ formation, we attempted a rescue experiment on apices overexpressing an inducible form of the *PECTIN METHYLESTERASE INHIBITOR3* gene (*PMEI3oe*); the PMEI3 enzyme acts to block HG de-methyl-esterification and rigidifies cell walls in the shoot apex [34]. As shown previously [20], [34], induced *PMEI3oe* lines display an organ-free *pin1*-like meristem upon induction (Fig. 2A). Local IAA applications on these naked meristems failed to induce organ formation (Fig. 2B, 72 h post application). IAA application on *pin1* meristems in the presence of the inducer (EtOH) stimulated organ formation normally (data not shown). These results establish that the inhibition of HG de-methyl-esterification blocks organ formation despite the local accumulation of auxin, thereby confirming the position of de-methyl-esterification of HG downstream of auxin in organ formation.

To test if IAA application on induced *PMEI3oe* naked apices could trigger changes in tissue mechanics even though no organs were formed, we measured mechanical properties of such apices ∼18 hours after application. No changes in rigidity were observed around the application site in either IAA treated or mock treated apices (Fig. 2C–E; Fig. S6). Together, these data point to a required downstream role for HG de-methyl-esterification in auxin induced tissue softening and organ emergence.

### Local HG de-methyl-esterification is sufficient for local tissue outgrowth, but not whole organ development, in the absence of functional auxin transport

**Figure 3:**
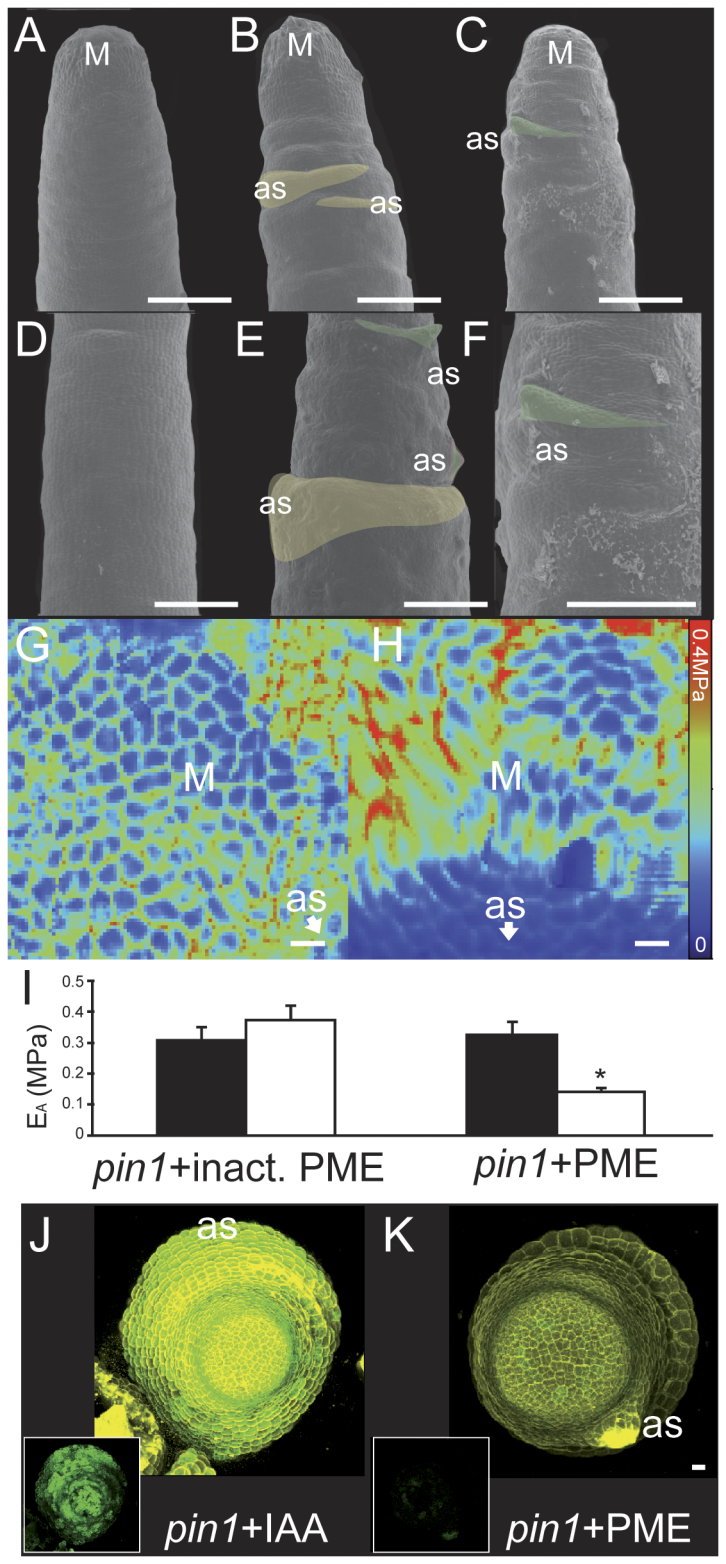
PME application on *pin1* meristems leads to tissue bulging and local tissue softening, but not functional organ development. SEM images of representative untreated (A) or PME treated *pin1* (B,C) meristems ∼72 h after treatment. Close ups of untreated meristem flank (D) or treated flank (E). (F) Direct magnification of the treated meristem in (C). Lateral stem bulging at the application site was observed on all treated plants (shaded yellow, n = 22), and stick-like lateral organs were observed in some samples (shaded green, n = 6/22). The two phenomena could be observed on the same stem (E). Young's modulus (E_A_, or 'rigidity') map of a representative *pin1* meristem treated with inactive PME (G) or active PME (H) as observed with a 5 µm spherical tip, ∼18 h post application. (I) Graphical display of averaged E_A_ data from PME treated (n = 6) or inactive PME treated (n = 3) meristems with values for meristem (black bars) and application site (white bars) (T-test on averages from n meristems: *pin1*-PME n = 3, p-value = 0.54; *pin1*+PME n = 6, p-value = 4.3E-3; significant difference at p-value<0.001, asterisk. Error bars are propagated standard deviations, statistics in Fig. S2). Non-averaged results for meristems displaying reduced rigidity can be found in Figure S7 (+Inactive PME n = 0/3, +IAA n = 3/3, +PME n = 5/6). (J) *DR5:GFP* signal (green) in a representative *pin1* meristem with IAA application, and (K) with PME application. Cell walls stained with propidium iodide (yellow). Insets in J–K show *DR5:GFP* signal alone. M: meristem, as: application site, Scale bars  = 100 µm (A–C), 50 µm (D–F) or 10 µm (G,H,J,K). *DR5:GFP* data for all meristems in Figure S8.

Next we tested if HG de-methyl-esterification alone could induce organ formation in the absence of auxin transport. We achieved local HG de-methyl-esterification on naked *pin1* meristems by applying PECTIN METHYLESTERASE (PME) -loaded beads; the PME enzyme acts to de-methyl-esterifiy HG. PME application triggered the formation of bumps or stick-like projections, but these did not develop further into functional lateral organs (Fig. 3B, 3C, 3E, 3F). No such bumps were observed upon application of denatured PME (Fig. 3A, 3D). Thus, in the absence of auxin transport, de-methyl-esterification of HG was only able to cause local tissue growth but not full organ formation.

We next examined the effect PME application on *pin1* apices had on cell wall mechanics. In concordance with induced local tissue growth, PME application led to local tissue softening as observed with a 5 µm spherical tip (Fig. 3H, 3I; Fig. S2, Fig. S7). No significant changes in rigidity were seen with mock application (Fig. 3G, 3I, Fig. S7) Finally, we examined whether PME application on *pin1* meristems was able to trigger a local auxin response, as visualized by the auxin signaling reporter DR5:GFP [9]. While IAA application on *pin1* apices triggered increase in auxin signaling (Fig. 3J, Fig. S8), PME application did not (Fig. 3K, Fig. S8). The *DR5* signal after PME application was similar to that in untreated apices (Fig. S8). In these experiments a 10× excess of IAA or PME were used to maximize the chance that a response could be visualized. Thus HG de-methyl-esterification was sufficient to induce tissue softening and tissue outgrowth; however, further development into a functional organ required auxin transport and a measureable auxin response.

### Coordinated local organization of the auxin transporter PIN1 is affected by inhibition of HG de-methyl-esterification

**Figure 4:**
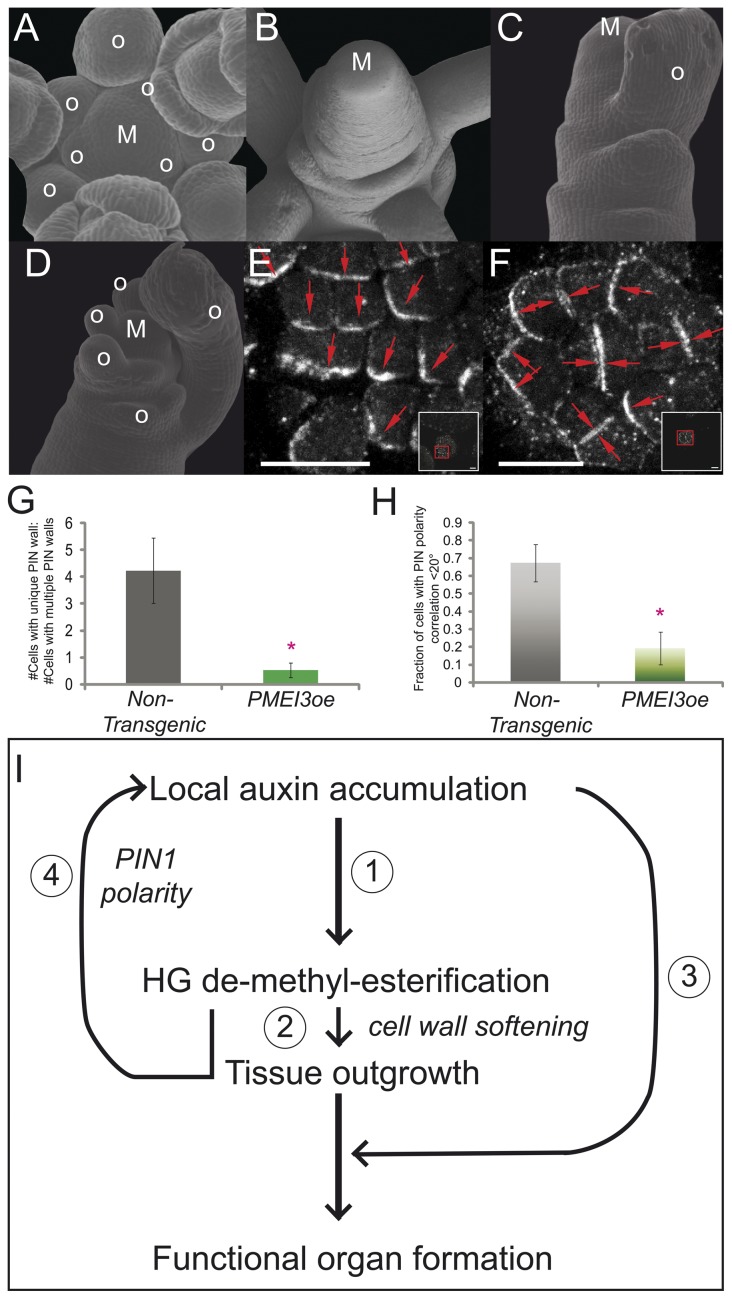
Recovery from inhibition of pectin de-methyl-esterification leads to altered organ size and phyllotaxis, and complete inhibition causes a disorganization in local PIN1 polarity. (A–D) SEM images of non-transgenic meristem (A), induced PMEI3oe meristem (B), or *PMEI3oe* meristems after 24 h induction and ∼72 h recovery (C,D). After recovery, organs present abnormal size (C) and phyllotactic positioning (D). Images representative of n = 100 meristems). (E–F) Immuno labeling of PIN1 protein in meristem epidermal cells of non-transgenic (E, as in A) or 24 h induced *PMEI3oe* (F, as in B) meristems. PIN1 displays local organization of polarity in non-transgenic meristems (E), but this organization is lost in *PMEI3oe* meristems (F). Red arrows indicate direction of PIN1 polarity within cells. Insets show larger section views for orientation (further details in Figure S8). (G) Quantification of PIN orientation within L1 cells as described by the ratio of cells showing unique wall polarity to those showing PIN1 on multiple walls (*NT* n = 482 cells, *PMEIi* n = 331 cells; sampled from 12 meristems per genotype). (H) Measurement of coordination of PIN1 polarity between adjacent cells as described by the fraction of neighbors exhibiting the same PIN1 orientation within 20° (*NT* n = 384 cells, *PMEIi* n = 286 cells; sampled from 12 meristems per genotype). T-test for significant difference was applied in both cases with n = above numbers, and a significance cut-off of p-value<0.001. (I) Model for the mechano-chemical regulatory loop underlying organ formation in plants: (1) Local auxin accumulation, driven by coordinated PIN1 polarity, leads to HG de-methyl-esterification. (2) HG de-methyl-esterification causes tissue softening (directly and indirectly) which then allows for tissue outgrowth; however, (3) local auxin accumulation is again required at the new organ to obtain a functional organ, (4) and this would be affected by PIN1 polarity - which is sensitive to tissue bulging and/or HG de-methyl-esterification. M: meristem, o: organ, Scale bars  = 100 µm (A–D) or 10 µm (E–F, including insets).

Since organ formation induced by de-methyl-esterification of HG required auxin transport, we next investigated a possible loop linking HG de-methyl-esterification and polar auxin transport. The existence of such a loop was suggested by the phenotype of recovering *PMEI3oe* apices; when *PMEI3oe* plants were allowed to recover from induction, the new organs did not follow the normal phyllotactic pattern and presented abnormal sizes (Fig. 4A, 4B vs. 4C, 4D). This phenotype is similar to that of plants recovering from chemical auxin transport inhibition [8], [40]. In contrast to these scenarios, *PMEI3oe* naked apices have functional auxin transporters. To test if the absence of HG de-methyl-esterification could alter auxin transport we immunolocalized PIN1 in *PMEI3oe* induced apices. In *PMEI3oe* lines, PIN1 presented disorganized polarity in the epidermis whereas in non-transgenic plants it presented areas of coordinated polar intracellular localization (Fig. 4E vs. 4F, Fig. S9). In *PMEI3oe* meristems, PIN1 could be observed in adjacent membranes of two neighboring cells (Fig. 4F), a phenomenon not seen in non-transgenic apices (Fig. 4E). Additionally, no PIN1 convergence points could be detected consistent with a lack of organ formation (Fig. S9). In order to more quantitatively examine PIN polarity, we measured the ratio of cells with a unique PIN carrying wall to those with multiple PIN carrying walls (Fig. 4G); In *PMEI3oe* plants this ratio was close to 1 indicating that most cells have multiple PIN walls. To look at PIN coordination between cells, we measured the fraction of neighboring cells displaying PIN orientation within 20° of a given reference cell (Fig. 4H). While non-transgenic apices displayed and average of ∼0.65 correlation, this was reduced to ∼0.2 in induced *PMEI3oe* apices. In conclusion, inhibition of HG de-methyl-esterification led to disruption of normal PIN1 polarity organization in the apex.

## Biological Discussion

Here we provide evidence that local accumulation of auxin in the shoot apex leads to tissue softening and, thus, organ outgrowth. For roughly 80 years a role for auxin in tissue softening has been known [41], [42]. Over time and with many experiments, it became clear that auxin induces changes in cell wall pH, cell wall mechanical properties, cell wall chemistry, and cell wall synthesis [25], [26], [27], [37], [38], [43], [44], [45], [46], [47], [48], [49], [50], [51]; however most of these experiments were performed on hypocotyl or coleoptile tissue. Here we demonstrate that auxin triggers changes in cell wall mechanics at the shoot apex, providing direct evidence for a long assumed link between auxin and new organ emergence.

Within the past 25 years several key components in auxin-mediated changes in cell mechanics have emerged, including the cell wall loosening expansins [17], [18], [52], [53], [54], Xyloglucan endotransglucosylase/hydrolases (XTHs/XETs)[55], [56], [57], and polygalacturaonases (PG). With respect to new organs, expansin expression is indicative of organ formation at the apex, and ectopic expansin activity can trigger organ formation [17], [18], [19]. Interestingly, the cell wall matrix is also critical as changes in the cell wall pectin matrix chemistry, the de-methyl-esterification of HG, are necessary and sufficient for new organ emergence at the shoot apex [20];these changes in pectin chemistry alter the elastic mechanical properties of the cell wall under nano-indentation [34]. Here, we demonstrate that auxin leads to tissue softening through the de-methyl-esterification of HG and that this chemical modification of the cell wall is required for auxin-induced organ formation. As we have previously discussed [32], the tissue softening associated with organ formation likely results from a combination of changes in the cell wall catalyzed by agents such as expansins, XET, PGs, and PME/PMEIs; however, it appears that the modification of the pectin matrix is either a major component of the measured softening or a required trigger (see Technical Discussion for more detail).

One of the most striking results of this study is that auxin signaling acts through a mechanical bottle-neck, namely de-methyl-esterification of HG. This implies that the complex suite of changes induced by auxin within the apex cannot proceed without HG-mediated changes in cell wall rigidity; there is evidence that pectin rigidity (as implied by de-methyl-esterification status) can limit the action of agents such as expansin in other tissues [58]. There also exists evidence that the selective methylation and de-methylation (methyltransfer) of existing pectins within the cell wall may be involved in cell expansion [37], [38]. Furthermore, calcium has been shown to inhibit elasticity in hypocotyls, again hinting at an important regulatory role for pectic complexes [27].

Another interesting observation arising from the work here is that physical modification of the wall mechanics alone via pectin, in the absence of functional auxin transport, was unable to yield a functional organ. These data imply that auxin action is required to trigger mechanical changes and *also* for developmental processes after initial mechanical bulging and/or other necessary mechanical changes, e.g. expansin activity (Schematic, Fig. 4I).

That blocking HG de-methyl-esterification disrupted organized PIN1 polarity indicates that mechanical changes within the apex may in turn control correct auxin distribution. This is supported by recent evidence that PIN1 can respond to changes in tissue mechanics [21], that PIN1 polarity requires cell wall integrity [23], and that changes in cell wall and membrane strain affect PIN1 polarity [24]. The induced *PMEIoe* naked apex had a functional auxin transport system, but it was disorganized- perhaps due to uniform wall mechanics, a lack of organs to act as organizers [6], a lack of differential growth which may organize PIN1 through tissue stresses [21], [22], or a combination of all. It is clear that upon release of PMEI activity, the apices regained competence to form organs although sizing and patterning was initially affected. These recovery phenotypes would be consistent with an apex with disorganized PIN1 being allowed to 'soften' due to auxin accumulation in random places, which then could feedback onto PIN polarity[21], [22], [23], [24] and stabilize organ size and phyllotaxis.

But what does the presence of this regulatory loop between auxin and tissue bulging mean? Phyllotactic patterning is extremely robust [59]; a mechano-chemical loop may provide a robust feedback mechanism that could help to control and buffer phyllotactic patterning at the apex (Fig. 4I). Since the auxin transporter PIN1 appears to respond to mechanical cues, it is also possible that the mechanical map of the meristem (areas of rigidity and softening) help to coordinate PIN1 polartiy and localization. As hypothesized previously [34], the wild-type meristem displays areas of softening that begin in subepidermal layers, but rapidly progress to the epidermis during organ growth- where PIN1 is localized. The altered strain that could result from such changes in rigidity, could in turn effect cell membrane strain/stress, and thus effect PIN1 polarity as suggested recently [24].

This type of patterning mechanism, requiring the active directional transport of an instructive chemical signal and the mechanical changes it induces, both invokes historical ideas and inspires future directions; indeed, further exploration of mechano-chemical regulatory loops in developmental biology will likely provide a rich landscape of interdisciplinary hypotheses [4], [5], [60].

## Technical Discussion

AFM-based nano indentation has only recently been applied to plant cells and tissues [34], [61], [62], [63], [64], as such many technical questions arise from its application (Note that we define nano indentation based upon the precision of the AFM vertical movement and the sub micrometer depth of indentations performed). We will attempt to discuss some of these points here (More discussion can be found in [32], [34]). First, what structural part of the cell wall contributes to the measured properties? For the scale of the experiments presented here, a large part of the data likely comes from the pectin matrix either directly or indirectly by influencing the behavior of embedded cellulose fibers. This is supported by immunocytochemistry and genetic manipulations [20], [34]. Thus, it appears as though the rigidity of the pectin matrix has a large influence on the patterning of growth. Discussion on how changes in pectin rigidity might influence cell growth mechanics may be found in [32], [33].

Second, are indentations perpendicular to the axis of growth informative? AFM tips indent tissues and cell wall segments perpendicular to the tissue surface, and in many tissues this is also perpendicular to the major growth axis. Based on the hypothesis that the rigidity data presented here is majorly influenced by the pectin matrix, it is likely that data perpendicular to the axis of growth is highly relevant; as a gel, the pectin matrix should behave as a relatively isotropic material under indentation and thus its properties perpendicular to the growth axis very close to those along it. In addition, within the meristem organ outgrowth will occur perpendicular to the surface as organ emergence is a plane-breaking phenomenon. As such, data on wall properties perpendicular to the surface may be highly relevant. As mentioned above, changes in the pectin rigidity may have significant effects on other cell wall polymers and their behavior. While our methods are not influenced by the predicted degree of cellulose anisotropy [34], other larger scale methods are [65] and a combination of techniques is required for a more complete understanding of growth mechanics.

Thirdly, if the above points are assumed, how could changes in the isotropic pectin matrix lead to localized anisotropic growth as seen in organ formation? It is possible that changing the matrix rigidity alters the elastic strain profile of the cell, a phenomenon which could be predicted to alter microtubule orientation and thus redirect cellulose orientation, yielding anisotropy [21], [22], [66]. This posits that a localized change in an isotropic material could yield anisotropic outgrowth via feed forward signaling. If we assume that not everything we measure is pectins, but also that a contribution for the important xyloglucans is detected, this framework still holds as it is unlikely that hemicelluloses display anisotropy independent of cellulose microfibrils.

Lastly, what happens when a curved surface is probed with a nano-indenter? For the most part, it is assumed during data interpretation that the indentation occurs normal to the material surface. This is obviously an over simplification. At any given position, the indenter tip will be at an angle to the sample, and the degree of the angle will be determined by the curvature of the sample- when the degree is large enough some of the energy in the system is lost leading to bias in the data due simply to geometry. In order to assess the bias introduced by sample geometry, we developed a new procedure: replicate meristems were produced from a silicon polymer which had meristem geometry but uniform mechanical properties. These types of samples enable the effects of geometry to be assessed. As shown in Figure S5, a silicon *pin1*meristem did show a bias due to geometry (Wilcoxon signed rank test, W = 8337.5 p-value<2.2E-16, mean percent difference 22%); however, the geometrical bias is dwarfed by the biological difference produced by IAA application (Wilcoxon signed rank test, W = 25421 p-value<2.2E-16, mean percent difference 128%). These experiments provide a concrete comparison method for analyzing the effect of sample geometry, and will hopefully contribute to the ongoing development of a precise analytical method for subtracting such a bias from data.

Within the past two years several research groups have begun using AFM to explore cell mechanics on tissue and single cell levels. This new and exciting application has already opened up new avenues of research, and as in this work, confirmed long assumed hypothesis. AFM-based nano indentation is a valuable tool for plant research, whose interpretation and development are continually evolving, providing new biological insights and advancing technological ideas.

## Materials and Methods

### Plant Material and Growth Conditions

*Arabidopsis thaliana* plants were grown on soil in controlled chambers under short-day conditions as described previously [20], unless otherwise indicated. *PMEI3oe* transgenic *Arabidopsis* plants were described previously [20]; briefly plants contained both *35S:alcR* and *alcA:PMEI3oe* transgenes allowing for widespread ethanol induction of *PMEI3*. Mutant *pin1* plants used were of the *pin1*–*7* allele in the Columbia background. Mutant *pin1*–*7* plants with the DR5:GFP construct were described previously [9], grown in culture on full MS media in long-day conditions, and observed just after bolting.

All experiments were performed on young primary inflorescence meristems just after bolting.

*PMEI3oe* transgenic *Arabidopsis* plants were grown on soil until just after bolting, and induced as follows: plants were placed with their pots inside plastic bags with one upper corner cut off to encourage air flow, within each pot a 0.5 mL microfuge tube was placed open containing roughly 100 µL of pure ethanol, and the plants were left over night for induction before observation.

### Application of modifying agents

Application of auxin (IAA) or PME were performed by loading silicon beads with either chemical as described previously [20]. For IAA application, 10 µM IAA or inactive IAA was loaded onto beads. Inactivated IAA was produced by overnight boiling of an active IAA solution, and demonstrated by a lack of organ inducing ability. For PME application, 0.01 U/ µL of PME enzyme in 10 mM PBS was loaded onto beads with an overnight incubation at room temperature. Beads were extracted from solution using forceps and placed upon meristems within the peripheral zone. Usually 1–2 beads were applied to a meristem. A schematic of bead position over time of assay can be seen in Figure S4. For *DR5:GFP* response in *pin1*–*7* mutant plants, PME and IAA were applied at 10× concentration to ensure any possible response would be seen (100 µM IAA and 0.1 U/ µl PME).

### Scanning Electron Microscopy

Images were obtained with an S-3500N variable-pressure scanning electron microscope (Hitachi) using a 5 mV vacuum and standard conditions. Scattered Electron and Back Scattered Electron images were collected.

### Confocal Microscopy

*A. thaliana pin1*–*7*/*DR5:GFP* meristems were treated with the appropriate chemical and imaged after ∼18 h (time of rigidity response but before IAA-triggered bulging). For imaging, meristems were dissected from plants and stained in 0.05% propidium iodide for 10 minutes (for visualization of cell walls). Confocal stacks were taken for x meristems per treatment (+PME n = 9,+IAA n = 8, *pin1* Controls n = 4, *WT* sibling Controls n = 5), using a 63× long-distance water immersion lens attached to a Leica DMR XE7 as described in [67]. Samples were imaged in water, and 0.5 µm deep optical sections were taken to cover the depth of the meristem and a significant portion of the flank. Images were collected in two channels: GFP and propidium iodide. Resulting image stacks were processed using Leica LAS AF software (v. 2.3.5) to provide maximum projections. Images in Figure 3 are representative of all samples examined (See Fig. S8 for images of all samples).

### Immuno-labeling of pectins and PIN1

Immuno-labeling of de-methyl-esterification of HG was conducted on 6 µm thick transverse sections, from 9 *pin1* inflorescence meristems using 2F4 antibodies in a buffer containing 0.5 mM CaCl_2_ in the presence of milk as described [68]. Immuno-labeling of PIN1 was conducted on transverse sections of 7 induced *PMEI3oe* (∼24 h) and 7 non-transgenic inflorescence meristems as described in [10]. Representative meristems with serial sections in Figure S9.

### AFM measurements

The AFM data were collected following the same protocol as previously described [34], except that the AFM machine, a stand-alone NanoWizard AFM, was now equipped with a CellHesion module allowing greater z-movement (JPK Instruments AG, Germany). Meristems were dissected from soil grown plants and immobilized on glass slides and surrounded by stiff agarose. Measurement of wall properties alone were ensured by suppression of turgor pressure by immersion of all meristems in a hypertonic solution a minimum of 30 minutes before measurement (0.55 M mannitol). We have previously demonstrated that this causes plasmolysis in mersitems [34]. The following numbers of meristems were analyzed: *pin1* (+ IAA n = 13, + inactive IAA n = 7, + PME n = 6, + denatured PME n = 3), 24–48 h induced *PMEI3oe* (+ IAA n = 9, + inactive IAA n = 11). When chemically-loaded beads were applied first to meristems, the beads were washed loose (or knocked loose) when meristems were prepared for AFM scanning, and the scans were made just apical to the bead position (See Fig. S4); Position of beads was noted. The following cantilevers were used: ‘Nano World’ (NanoWorld AG Headquarters, Neuchâtel, Switzerland) TL-NCH-20 tips with a spring constant of 10–130 N/m (those used were estimated to be 1.5 N/m) with Sphere Tips of a 900–1100 nm radius or tip-less probes. Tip-less probes were mounted with 5 µm borosilicate beads attached with Araldite glue (Bostik SA. 77 170, Coubert France). All force spectroscopy experiments was performed as previously described [34]; briefly, rigidity of samples was determined as follows: an AFM cantilever loaded with a spherical tip was used to indent the sample over a 60×60 µm square area, indentations were kept to <10% of cell height (∼250–500 nm), within the area 64×64 measurements were made resulting in 4096 force-indentation experiments, each force-indentation experiment was treated with a Hertzian indentation model to extrapolate the apparent Young's modulus, each pixel in a rigidity map represents the Young's modulus from one force-indentation point. For topographical reconstructions, height of each point was determined by the point-of-contact from the force-indentation curve; each contact point is from the same curve used to determine E_A_. Rigidity data was projected on to topographical maps using MatLab.

### Apparent Young's modulus calculations

The Young's modulus is a parameter that relates applied force to indentation; in stricter terms it is the ratio of uniaxial strain and uniaxial stress, within a liner elastic behavior. In order to ensure that indentations are performed within a linear elastic range the following technical controls are confirmed in all tissues used: 1) Indentation depth is <10% of total cell height, 2) approach and retraction curves from the experiments are examined for hysteresis [32], and 3) indentation times are 0.2 s total to avoid viscous deformation.

In contrast to our previous work [34] the Apparent Young's modulus in this study was calculated using the JPK Data Processing software (ver. spm-4.0.23, JPK Instruments AG, Germany), which allows for a more standardized analysis (although possibly less accurate in certain situations).The Young's modulus is estimated using a standard Hertzian contact model for spherical indenters. The switch in analysis method was deliberately performed in order to allow greater comparability between different labs using AFM-based technologies to study mechanics. Only the approach curve was used in our analysis to avoid any adhesion interference. The best fit was obtained using a Hertzian model with 0.5 µm or 2.5 µm as tip radii, for a cantilever loaded with the 5 µm or 1 µm spherical beads respectively. A Poisson ratio of 0.5 was assumed for the material. For graphed data, 30–40 points per area of interest were selected (as randomly as humanly possible) and averaged, for each meristem. For 'mean of mean' graphs standard propagation of error calculations were applied. A standard t-test was applied to test for differences between treatments.

### PIN1 Orientation Measurements

PIN1 orientation was measured using ImageJ freeware (ImageJ 1.43 u Wayne Rasband NIH, USA http://rsb.info.nih.gov/ij). The PIN1 orientation for a cell was chosen as the cell wall with the highest level of immunoreactivity. This orientation was then compared to the average orientation of two neighboring cells which presented the closest centers to the reference cell.

## Supporting Information

Figure S1 **Rigidity of pin1 meristems after IAA application as measured with 5 µm and 1 µm tips.** Changes in rigidity for pin1 meristems treated with inactive- or active-IAA loaded beads as measured with a 5 µm shperical tip (A) or a 1 µm spherical tip (B). Black bars are data from meristem, white bars are data from application site. Each set of black/white bars represents an average of 50–100 data points from a single meristem. At the begining of each graph, mean values for all points of all meristem/application site values are displayed. Asterisk idicates when meristem is significantly more rigid than the application site (P< 0.01). Note that 3/7 meristems show significant softening with the 5 µm tip after innactive IAA application, although this does not affect the average data. See Figure S2 for details of statistical results.
(TIF)

Figure S2 **Sample numbers and statistical results for all AFM data in supplemental information.** For Figures S1, S4, and S7: Significance was determined as TRUE for a reduced rigidity in the ‘periphery’ compared to ‘meristem’ when p<0.001. Mean data is a mean of means from the listed data below that entry, with standard propagation of error applied. For single meristem data, N refers to the number of EA values taken from that meristem, evenly distributed between relevant location areas. P-values were determined by a Student's T-test in Microsoft Excell. For Figure S5: Wilcoxon signed rank tests were applied to these data, which were determined to be non-normal by a Shapiro-Wilks test. Significance was determined as TRUE for a reduced rigidity in the ‘bottom area’ compared to the ‘top area’ when p<0.001. N refers to the number of EA values taken from that meristem/cast, evenly distributed between the relevant physical locations.
(TIF)

Figure S3 **Control experiments for** **Figure 1** **.** (A) Inactive IAA does not trigger organ formation on a mutant inflorescence apex (t = 48 h post application). Apparent Young's modulus (EA, or 'rigidity') map of a representative pin1 meristem ∼18 hours post inactive IAA application as determined with a 1 µm (B) or 5 µm (C) spherical tip. Total number of meristems analyzed - IAA (n = 6). (D) Graphical display of averaged EA data from all meristems with values for meristem (black bars) and application site (white bars). (E,F) Topographical reconstruction of measured surfaces, as estimated by AFM point-of-contact, with the rigidity maps of (B,C) respectively used to color the surface. (G) 2F4 labeling of HG de-methyl-esterification in a representative pin1 meristem ∼ 18 hours after inactive IAA application (n = 9). M: meristem, as: application site, Scale bars  =  100 micron (A,G) or 10 micron (B,C).
(TIF)

Figure S4 **Schematic of chemically-loaded bead application and kinetics, and position of AFM reads.** (1) Bead application site at t = 0 h, (2) bead position at t = ∼18 h, (3) bead position at t∼48 h. Red square indicates area of AFM read at t = ∼18 h; M  =  meristem as in AFM scans at t = ∼18 h, and as =  position of application site just below the position of AFM read at t = ∼18 h. As such, AFM reads are just above t = ∼18 h bead position, to negate any mechanical effect of the bead itself.
(TIF)

Figure S5 **Effect of sample geometry on EA values.** To examine the effect on sample geometry on the rigidity data (presented as apparent Young's Modulus, EA) data obtained from a ‘fake’ silicon pin mutant meristem (A), data obtained from an untreated pin mutant meristem (B), and data obtained from an IAA applied pin mutant meristem (C) were compared. Within each panel are a topographical height map, an EA map projected on the topographic surface. (D) Boxplot of regional EA values corresponding to boxes on the height map, distributions were compared with a Wilcoxon Signed Rank Test and all differences between ‘top’ and ‘bottom’ areas were significant at p-value <0.001 (n per box  =  200 (silicon), 160 (pin meristems); pink asterisks; all distributions were non-normal as determined by a Shapiro-Wilks test except for the +IAA bottom area); however, the percent difference of the control samples was dwarfed by that in the +IAA experimental condition. To maximize the possibility of discovering geometry induced error, the silicon pin meristem was imaged with a new scan set-up allowing X:Y:Z dimensions of 100∶100∶25 µm; thus the silicon meristem presented displays larger analyzed curvature than any plant sample in this study. (A) The silicon meristem EA map shows little bias due to geometry as seen in the EA map and the graph of regional values (D, %diff = 21.86); interestingly the flatter top region appears slightly less rigid than the sloped area. (B) The control pin meristem without IAA application also shows very slight EA bias due to geometry as seen in the EA map and the regional graph; here the predicted decrease in rigidity on sloped areas is observed, although slight (D, %diff = 14.81). (C) For the experimental pin meristem with IAA application, the difference between the area proximal to the application site (AS) and the non-exposed ‘top’ area of the meristem is striking and far larger in magnitude than that expected by geometrical bias alone (D, %diff = 128.41 vs. 14–22% for controls). As such, while an appropriate data-based correction method for geometrical bias is under development- within the experiments presented in this paper the experimental/biological differences eclipse those due to geometrical bias. See Figure S2 for details of statistical tests.spherical tip.
(TIF)

Figure S6 **Rigidity of PMEI3oe meristems after treatment with IAA.** Rigidity for PMEI3oe meristems treated with inactive- or active-IAA loaded beads as measured with a 5 µm spherical tip. Black bars are data from meristem, white bars are data from application site. Each set of black/white bars represents an average of 50–200 data points from a single meristem. At the beginning of each graph, mean values for all points of all meristem/application site values are displayed. Asterisk indicates when meristem is significantly more rigid than the application site (P< 0.01). Note that 3/20 meristems show significant softening after application, although this does not affect the average data. See Figure S2 for details of statistical tests.
(TIF)

Figure S7 **Rigidity of pin1 meristems treated with IAA, PME, or Inactive PME.** Rigidity for pin1 meristems treated with inactive-IAA, active-PME, or PME loaded beads as measured with a 5 µm spherical tip. IAA-treated meristems serve as a control for decreased rigidity. Black bars are data from meristem, white bars are data from application site. Each set of black/white bars represents an average of 50–200 data points from a single meristem. At the beginning of each graph, mean values for all points of all meristem/application site values are displayed. Asterisk indicates when meristem is significantly more rigid than the application site (P< 0.01). Note that 1/6 meristems did not show significant softening after PME application, although this does not affect the average data. See Figure S2 for details of statistical tests.
(TIF)

Figure S8 **DR5:GFP signal in pin1 mutants treated with IAA, PME, or untreated DR5:GFP signal in pin1 mutant meristems with (A) IAA application, (B) PME application, or (C) no application.** Yellow channel  =  propidium iodide cell wall staining, Green channel  =  DR5:GFP. Note that some meristems experienced drying during the experiment which can be seen are large areas of propidium iodide staining (pink asterisks). Also note that several meristems did not stain well with propidium iodide (blue asterisks). Beads often washed off during confocal preparation and as such are only occasionally visible.
(TIF)

Figure S9 **Serial sections in non-transgenic and induced PMEI3oe meristems with PIN1 immunolocalization.** Serial transverse sections through representative non-transgenic (A–C) and induced PMEI3oe (D–F) meristems, showing PIN1 immunolocalization. Red boxes in (A) and (D) indicate areas shown in Figure 4E, 4F. Asterisk is (B) indicates PIN1 polarization in subepidermal layers involved in vein formation; no such organization is seen in the subepidermal tissues of PMEI3oe meristem although there appears to be more PIN1 in subepidermal tissues in more locations (E,F). Scale bars  =  10 µm.
(TIF)
