# Supplementary figures and images for: Mechano-Chemical Aspects of Organ Formation in Arabidopsis thaliana: The Relationship between Auxin and Pectin
Source: PLoS One. 2013 Mar 12;8(3):e57813. doi: 10.1371/journal.pone.0057813 (PMC3595255; doi:10.1371/journal.pone.0057813)

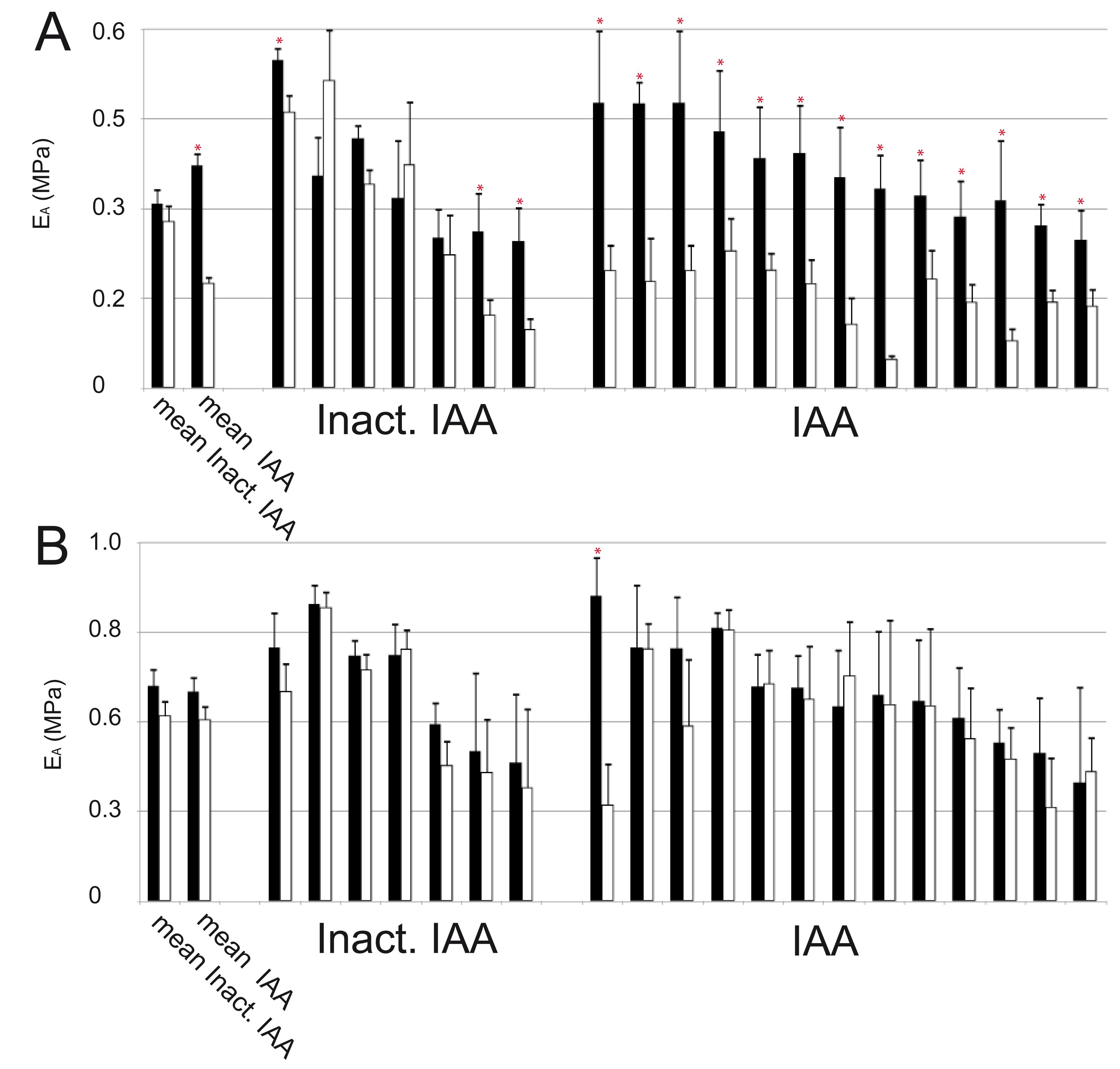

Supplement: Figure S1 — Rigidity of pin1 meristems after IAA application as measured with 5 µm and 1 µm tips. Changes in rigidity for pin1 meristems treated with inactive- or active-IAA loaded beads as measured with a 5 µm shperical tip (A) or a 1 µm spherical tip (B). Black bars are data from meristem, white bars are data from application site. Each set of black/white bars represents an average of 50–100 data points from a single meristem. At the begining of each graph, mean values for all points of all meristem/application site values are displayed. Asterisk idicates when meristem is significantly more rigid than the application site (P< 0.01). Note that 3/7 meristems show significant softening with the 5 µm tip after innactive IAA application, although this does not affect the average data. See Figure S2 for details of statistical results. (TIF) [file pone.0057813.s001.tif]

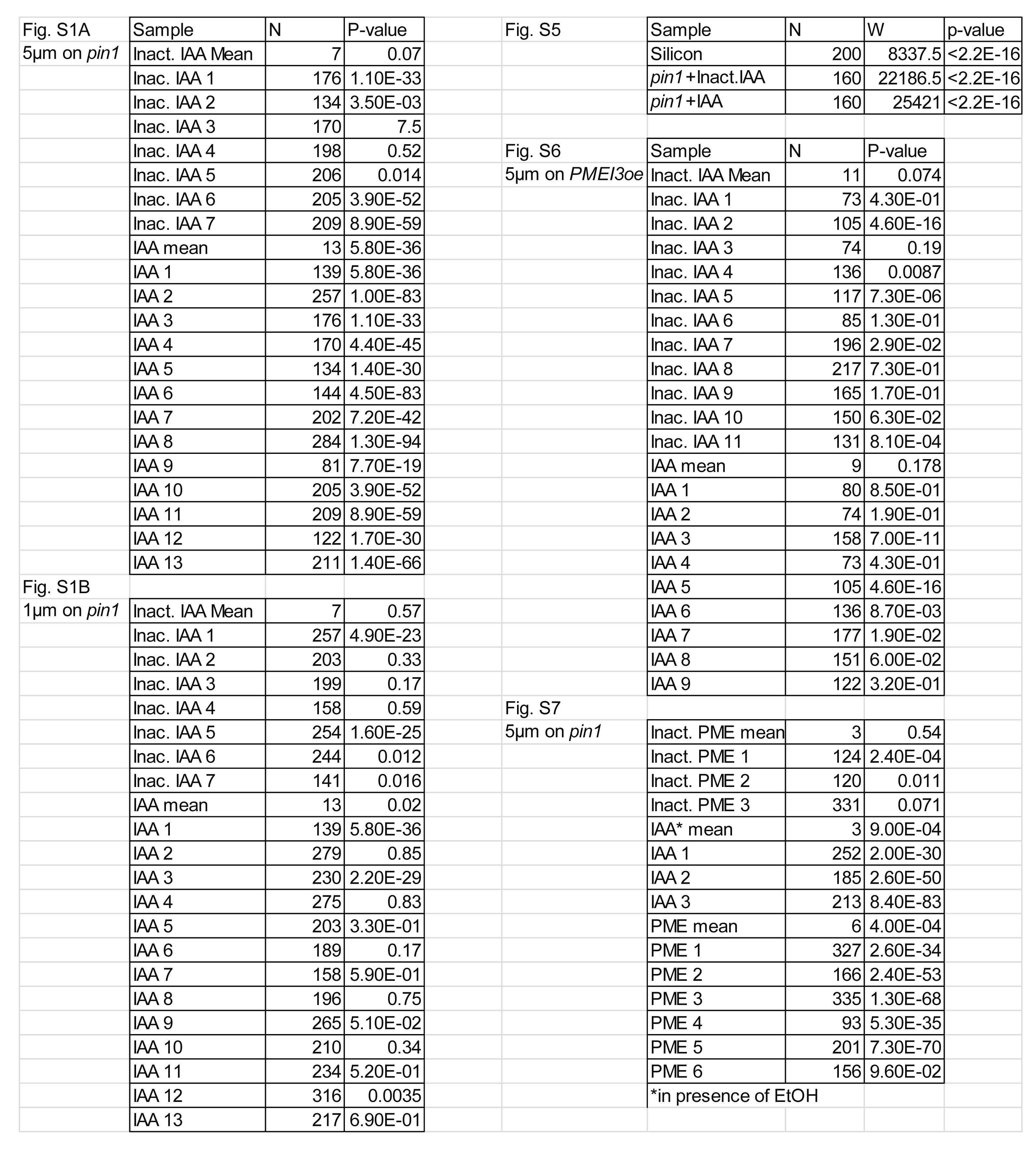

Supplement: Figure S2 — Sample numbers and statistical results for all AFM data in supplemental information. For Figures S1, S4, and S7: Significance was determined as TRUE for a reduced rigidity in the ‘periphery’ compared to ‘meristem’ when p<0.001. Mean data is a mean of means from the listed data below that entry, with standard propagation of error applied. For single meristem data, N refers to the number of EA values taken from that meristem, evenly distributed between relevant location areas. P-values were determined by a Student's T-test in Microsoft Excell. For Figure S5: Wilcoxon signed rank tests were applied to these data, which were determined to be non-normal by a Shapiro-Wilks test. Significance was determined as TRUE for a reduced rigidity in the ‘bottom area’ compared to the ‘top area’ when p<0.001. N refers to the number of EA values taken from that meristem/cast, evenly distributed between the relevant physical locations. (TIF) [file pone.0057813.s002.tif]

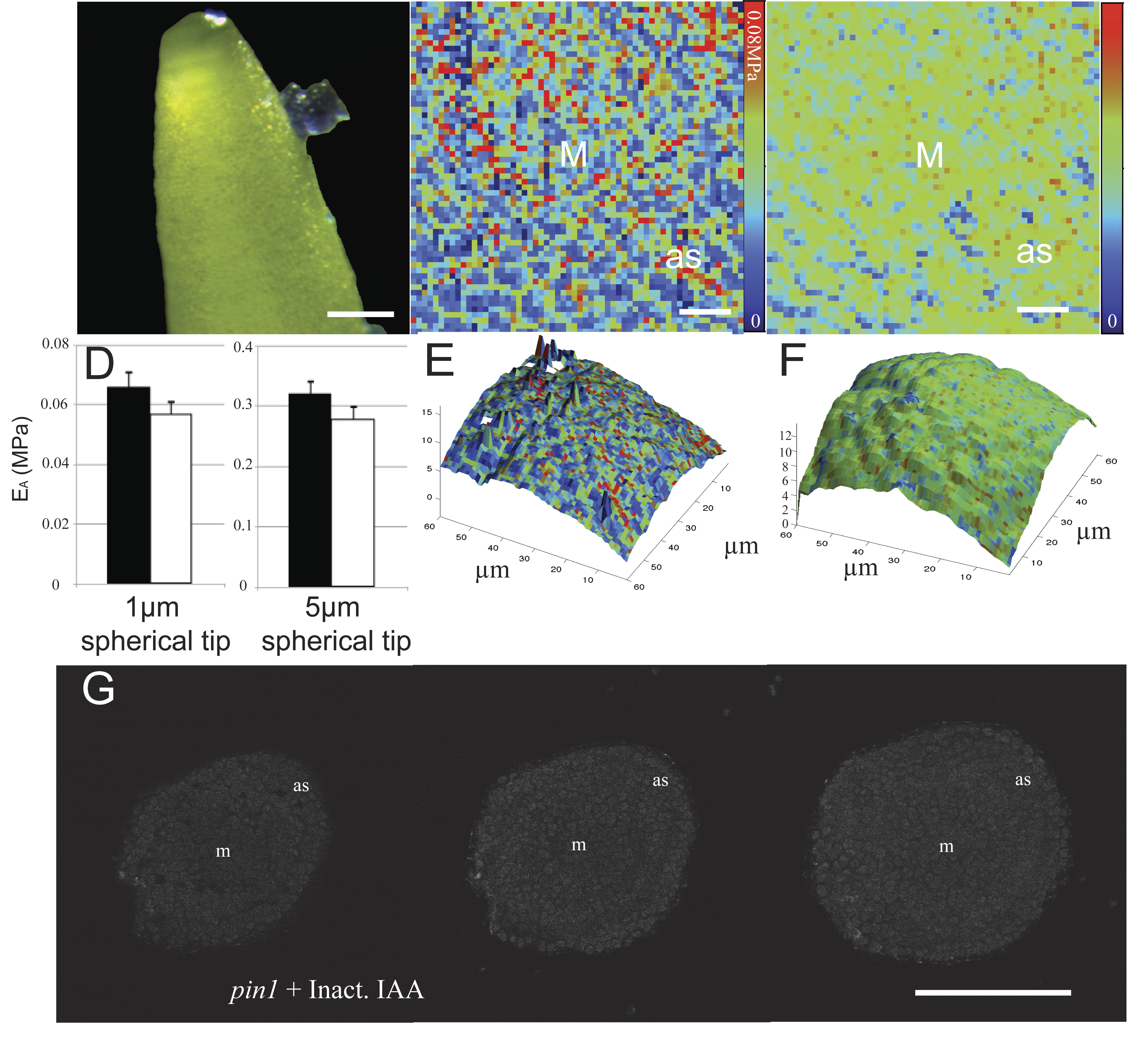

Supplement: Figure S3 — Control experiments for Figure 1 . (A) Inactive IAA does not trigger organ formation on a mutant inflorescence apex (t = 48 h post application). Apparent Young's modulus (EA, or 'rigidity') map of a representative pin1 meristem ∼18 hours post inactive IAA application as determined with a 1 µm (B) or 5 µm (C) spherical tip. Total number of meristems analyzed - IAA (n = 6). (D) Graphical display of averaged EA data from all meristems with values for meristem (black bars) and application site (white bars). (E,F) Topographical reconstruction of measured surfaces, as estimated by AFM point-of-contact, with the rigidity maps of (B,C) respectively used to color the surface. (G) 2F4 labeling of HG de-methyl-esterification in a representative pin1 meristem ∼ 18 hours after inactive IAA application (n = 9). M: meristem, as: application site, Scale bars = 100 micron (A,G) or 10 micron (B,C). (TIF) [file pone.0057813.s003.tif]

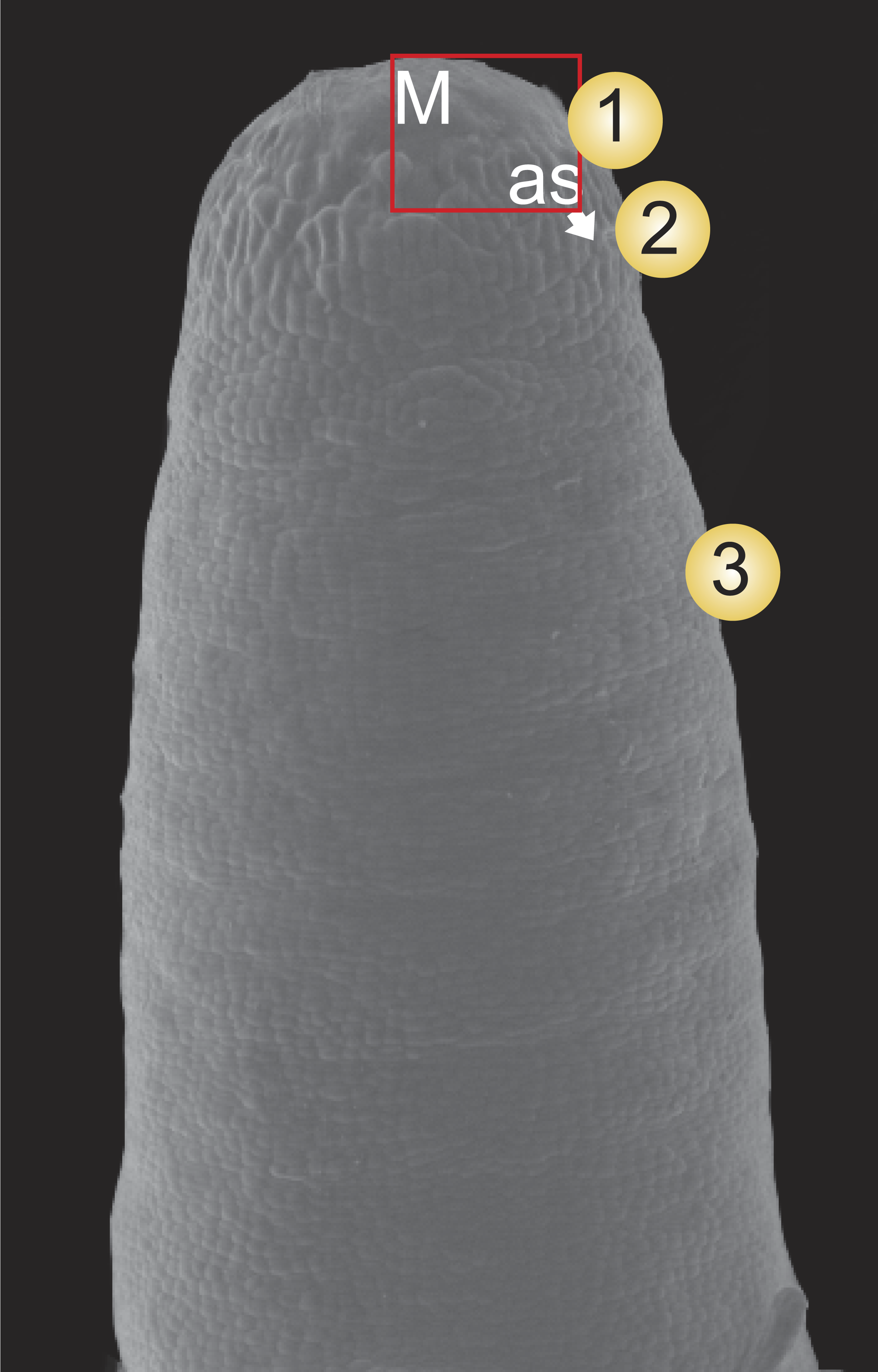

Supplement: Figure S4 — Schematic of chemically-loaded bead application and kinetics, and position of AFM reads. (1) Bead application site at t = 0 h, (2) bead position at t = ∼18 h, (3) bead position at t∼48 h. Red square indicates area of AFM read at t = ∼18 h; M = meristem as in AFM scans at t = ∼18 h, and as = position of application site just below the position of AFM read at t = ∼18 h. As such, AFM reads are just above t = ∼18 h bead position, to negate any mechanical effect of the bead itself. (TIF) [file pone.0057813.s004.tif]

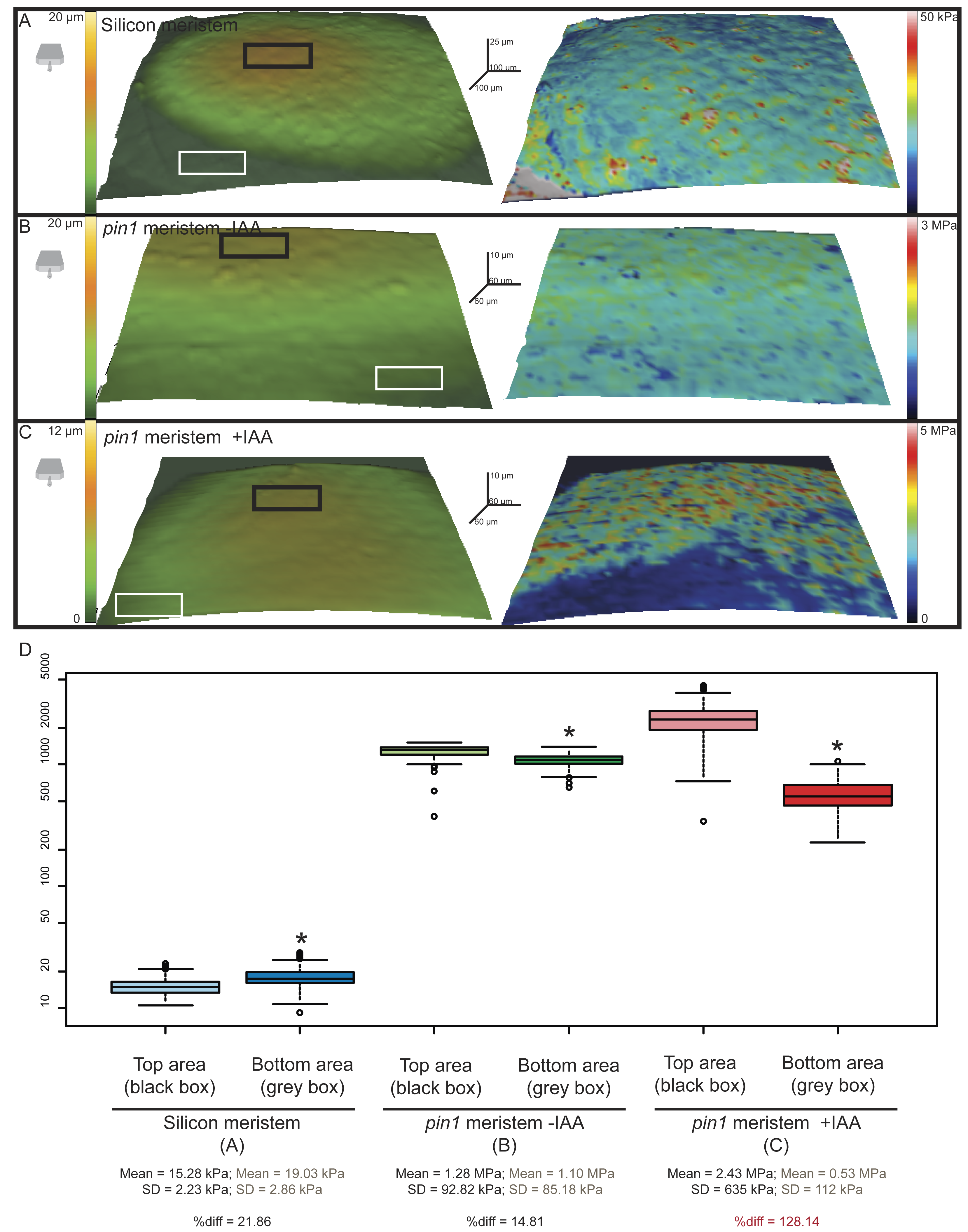

Supplement: Figure S5 — Effect of sample geometry on EA values. To examine the effect on sample geometry on the rigidity data (presented as apparent Young's Modulus, EA) data obtained from a ‘fake’ silicon pin mutant meristem (A), data obtained from an untreated pin mutant meristem (B), and data obtained from an IAA applied pin mutant meristem (C) were compared. Within each panel are a topographical height map, an EA map projected on the topographic surface. (D) Boxplot of regional EA values corresponding to boxes on the height map, distributions were compared with a Wilcoxon Signed Rank Test and all differences between ‘top’ and ‘bottom’ areas were significant at p-value <0.001 (n per box = 200 (silicon), 160 (pin meristems); pink asterisks; all distributions were non-normal as determined by a Shapiro-Wilks test except for the +IAA bottom area); however, the percent difference of the control samples was dwarfed by that in the +IAA experimental condition. To maximize the possibility of discovering geometry induced error, the silicon pin meristem was imaged with a new scan set-up allowing X:Y:Z dimensions of 100∶100∶25 µm; thus the silicon meristem presented displays larger analyzed curvature than any plant sample in this study. (A) The silicon meristem EA map shows little bias due to geometry as seen in the EA map and the graph of regional values (D, %diff = 21.86); interestingly the flatter top region appears slightly less rigid than the sloped area. (B) The control pin meristem without IAA application also shows very slight EA bias due to geometry as seen in the EA map and the regional graph; here the predicted decrease in rigidity on sloped areas is observed, although slight (D, %diff = 14.81). (C) For the experimental pin meristem with IAA application, the difference between the area proximal to the application site (AS) and the non-exposed ‘top’ area of the meristem is striking and far larger in magnitude than that expected by geometrical bias alone (D, %diff = 128.41 vs. 14–22% for c [file pone.0057813.s005.tif]

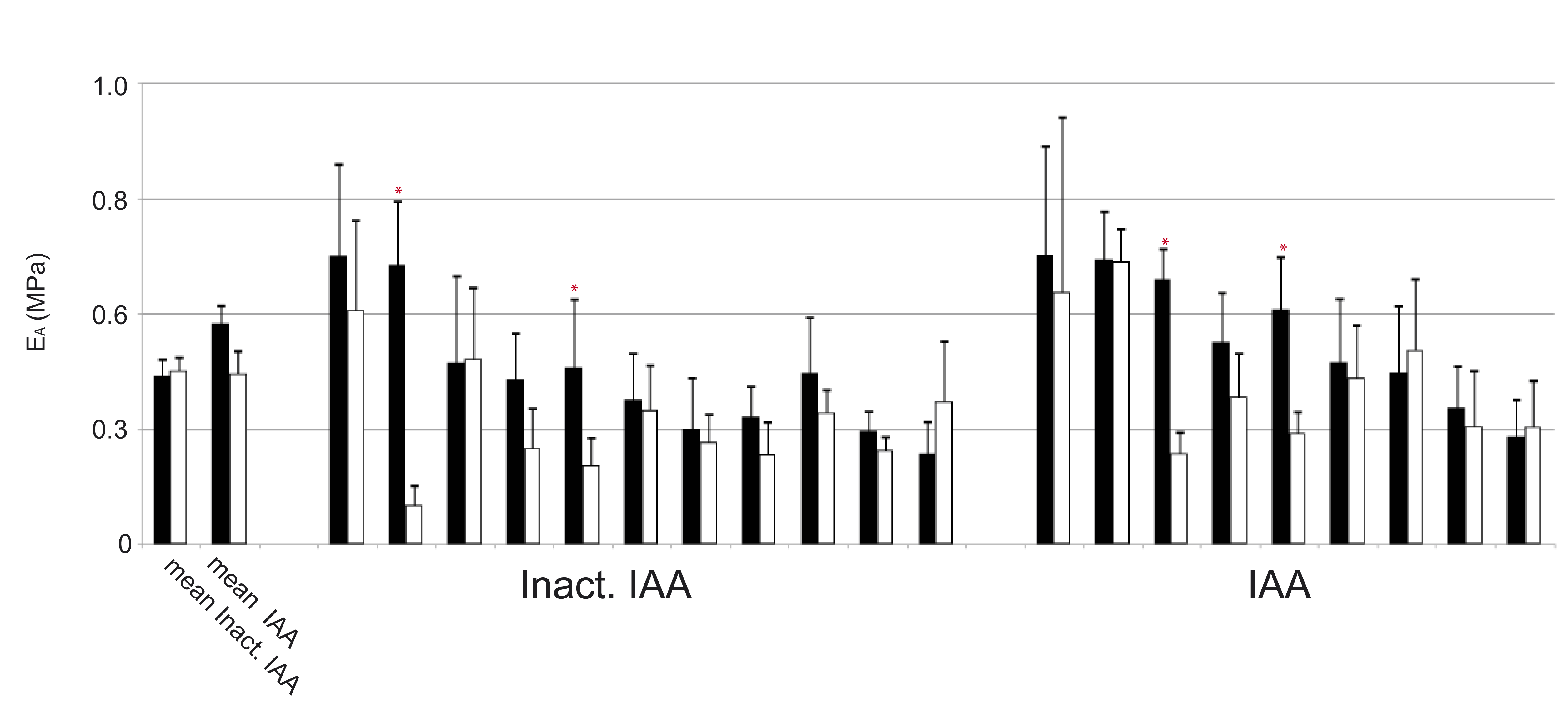

Supplement: Figure S6 — Rigidity of PMEI3oe meristems after treatment with IAA. Rigidity for PMEI3oe meristems treated with inactive- or active-IAA loaded beads as measured with a 5 µm spherical tip. Black bars are data from meristem, white bars are data from application site. Each set of black/white bars represents an average of 50–200 data points from a single meristem. At the beginning of each graph, mean values for all points of all meristem/application site values are displayed. Asterisk indicates when meristem is significantly more rigid than the application site (P< 0.01). Note that 3/20 meristems show significant softening after application, although this does not affect the average data. See Figure S2 for details of statistical tests. (TIF) [file pone.0057813.s006.tif]

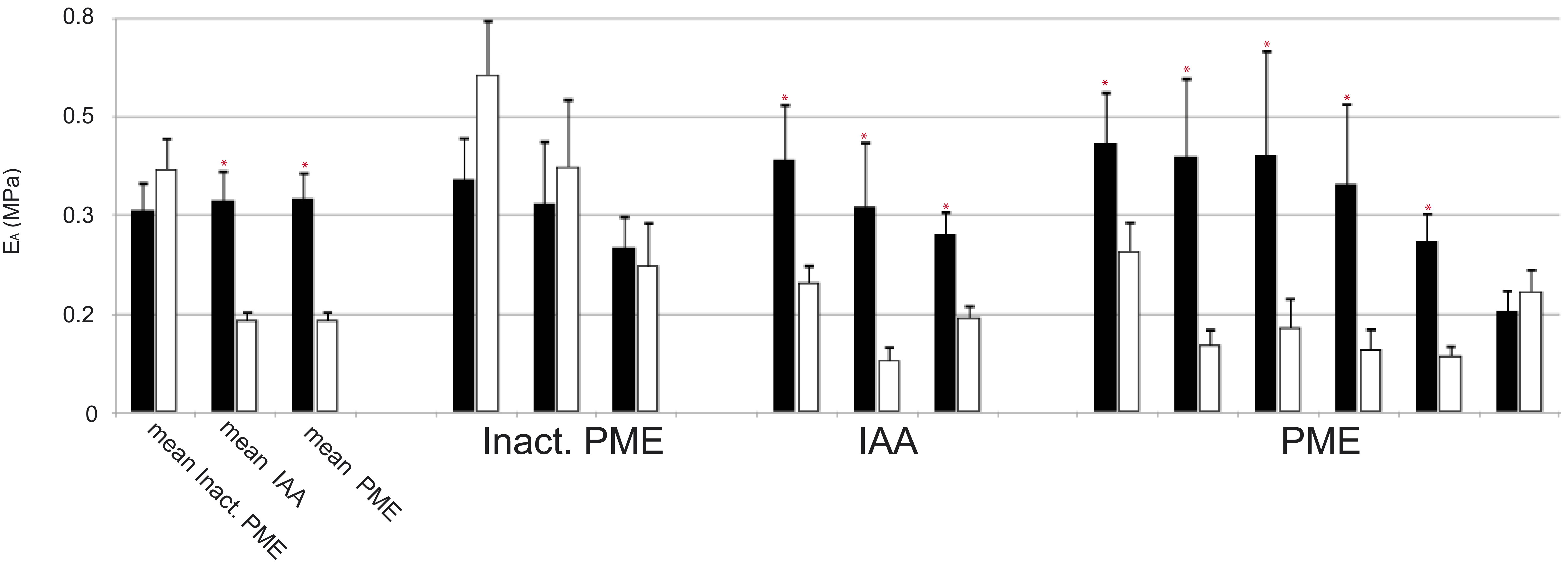

Supplement: Figure S7 — Rigidity of pin1 meristems treated with IAA, PME, or Inactive PME. Rigidity for pin1 meristems treated with inactive-IAA, active-PME, or PME loaded beads as measured with a 5 µm spherical tip. IAA-treated meristems serve as a control for decreased rigidity. Black bars are data from meristem, white bars are data from application site. Each set of black/white bars represents an average of 50–200 data points from a single meristem. At the beginning of each graph, mean values for all points of all meristem/application site values are displayed. Asterisk indicates when meristem is significantly more rigid than the application site (P< 0.01). Note that 1/6 meristems did not show significant softening after PME application, although this does not affect the average data. See Figure S2 for details of statistical tests. (TIF) [file pone.0057813.s007.tif]

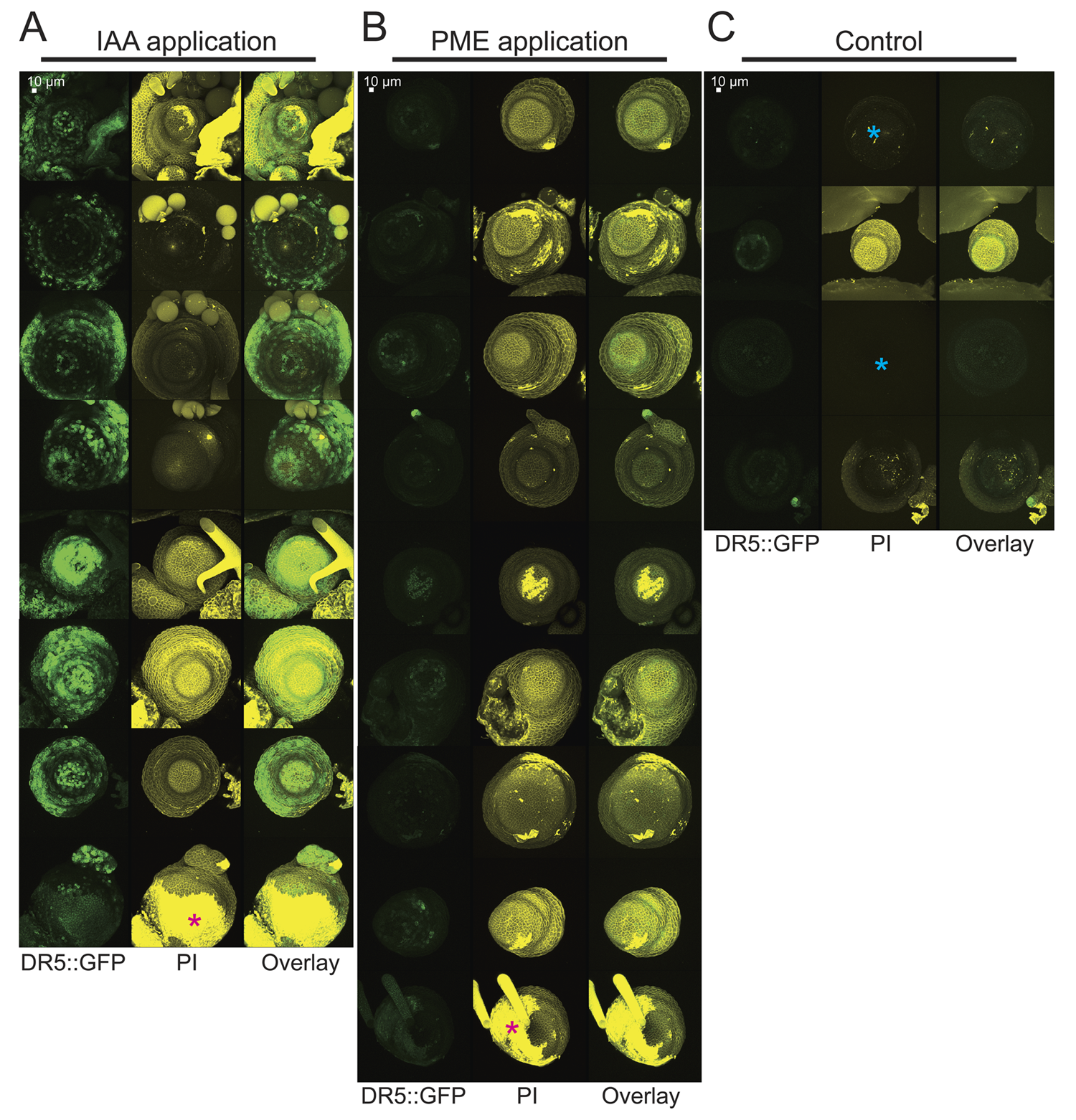

Supplement: Figure S8 — DR5:GFP signal in pin1 mutants treated with IAA, PME, or untreated DR5:GFP signal in pin1 mutant meristems with (A) IAA application, (B) PME application, or (C) no application. Yellow channel = propidium iodide cell wall staining, Green channel = DR5:GFP. Note that some meristems experienced drying during the experiment which can be seen are large areas of propidium iodide staining (pink asterisks). Also note that several meristems did not stain well with propidium iodide (blue asterisks). Beads often washed off during confocal preparation and as such are only occasionally visible. (TIF) [file pone.0057813.s008.tif]

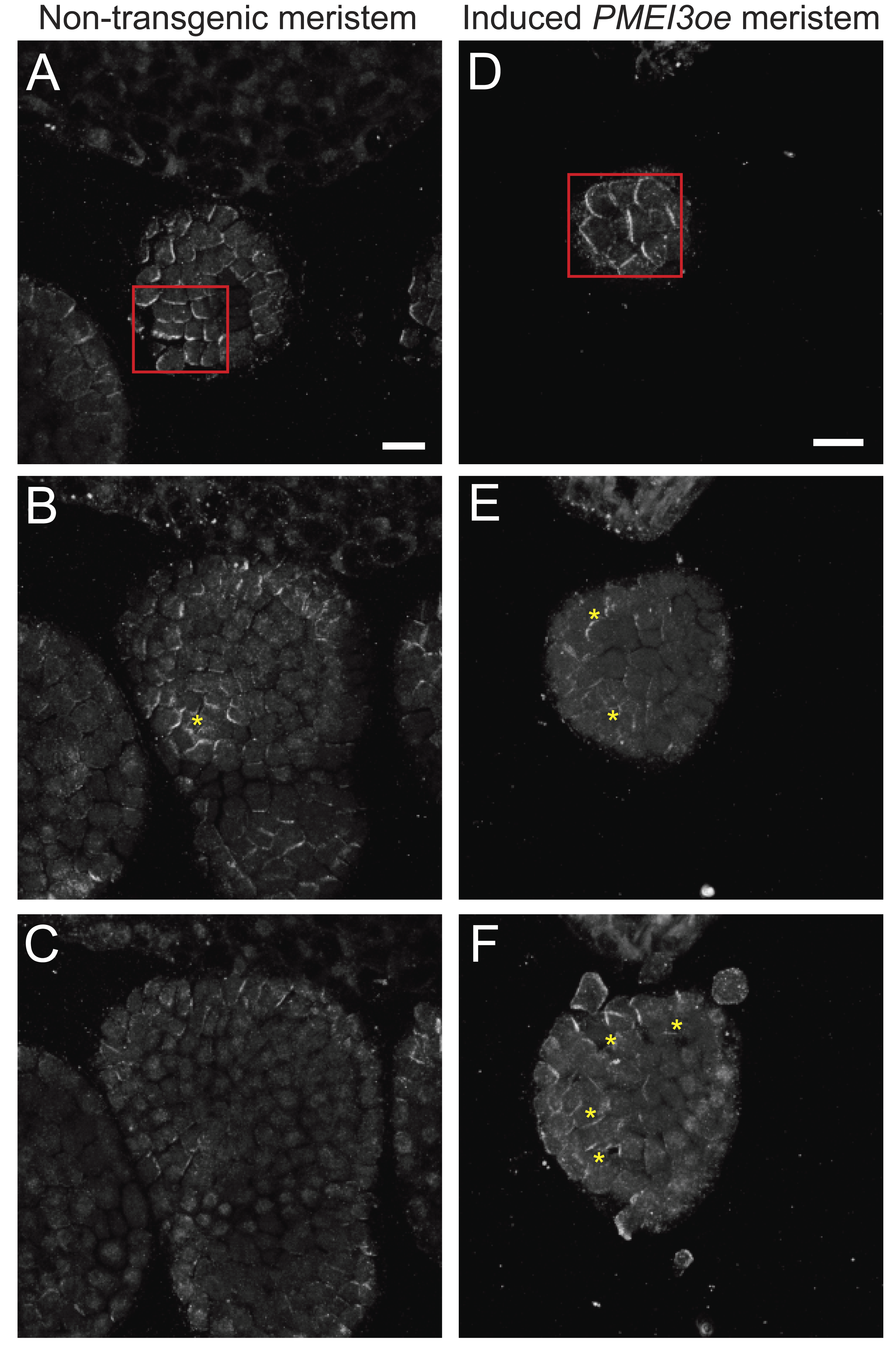

Supplement: Figure S9 — Serial sections in non-transgenic and induced PMEI3oe meristems with PIN1 immunolocalization. Serial transverse sections through representative non-transgenic (A–C) and induced PMEI3oe (D–F) meristems, showing PIN1 immunolocalization. Red boxes in (A) and (D) indicate areas shown in Figure 4E, 4F. Asterisk is (B) indicates PIN1 polarization in subepidermal layers involved in vein formation; no such organization is seen in the subepidermal tissues of PMEI3oe meristem although there appears to be more PIN1 in subepidermal tissues in more locations (E,F). Scale bars = 10 µm. (TIF) [file pone.0057813.s009.tif]
